# Cod-Liver Oil with Quinine

**Published:** 1856-02

**Authors:** John Horsley

**Affiliations:** The Laboratory, Cheltenham


### *Cod-Liver Oil with Quinine.*

[To the Editor of the Medical Times and Gazette.]

Sir,—I had thought that most chemists were aware that pure quinia was soluble in oils and spirits. Cod-liver oil with quinine, which at the present time seems to attract so much attention, and to have so many claimants for its discovery, is easily prepared as follows:—

First prepare the quinia by dissolving two drachms of the disulphate in half a pint of water, acidulated with two drachms of dilute sulphuric acid; then precipitate, by the addition of one and a half drachm of liquor ammoniæ, project and filter, and wash the precipitate with four or five ounces more water. When the precipitate has been well drained, sweep off the quinia, and introduce it into an evaporating pan and set it over a steam bath. The heat will cause the quinia to melt and separate from the interstitial water, which should be poured off, and the quinia collected on a piece of blotting paper to dry, when it should be powdered and preserved for use. To prepare the oil take sixteen grains of the quinia and dissolve in eight ounces of oil by means of a gentle heat. The oil will become slightly colored. This, however, is best obviated by dissolving the quinia in a mixture of one drachm each of ether and alcohol, and mixing the solution with the oil, and then evaporating the spirit by means of a steam bath, agitating it occasionally with a glass rod. If the oil requires filtration it should be passed through a piece of lint loosely inserted in the neck of a funnel. Every ounce of the oil will contain two grains of quinia. I am, etc.

JOHN HORSLEY.

The Laboratory, Cheltenham, Nov. 19, 1855.

